# COVID-19 Related Fears of Patients Admitted to a Psychiatric Emergency Department during and Post-Lockdown in Switzerland: Preliminary Findings to Look Ahead for Tailored Preventive Mental Health Strategies

**DOI:** 10.3390/medicina57121360

**Published:** 2021-12-13

**Authors:** Alessandra Costanza, Laura Macheret, Aline Folliet, Andrea Amerio, Andrea Aguglia, Gianluca Serafini, Paco Prada, Guido Bondolfi, François Sarasin, Julia Ambrosetti

**Affiliations:** 1 Faculty of Medicine, University of Geneva (UNIGE), 1206 Geneva, Switzerland; 2 Emergency Psychiatric Unit (UAUP), Department of Emergency and Department of Psychiatry, Geneva University Hospitals (HUG), 1211 Geneva, Switzerland; laura.macheret@hcuge.ch (L.M.); aline.folliet@hcuge.ch (A.F.); julia.ambrosetti@hcuge.ch (J.A.); 3 Department of Neuroscience, Rehabilitation, Ophthalmology, Genetics, Maternal and Child Health (DINOGMI), Section of Psychiatry, University of Genoa, 16132 Genoa, Italy; andrea.amerio@unige.it (A.A.); andrea.aguglia@unige.it (A.A.); gianluca.serafini@unige.it (G.S.); 4 IRCCS Ospedale Policlinico San Martino, 16132 Genoa, Italy; 5 Department of Psychiatry, Service of Liaison Psychiatry and Crisis Intervention (SPLIC), Geneva University Hospitals (HUG), 1211 Geneva, Switzerland; paco.prada@hcuge.ch (P.P.); guido.bondolfi@hcuge.ch (G.B.); 6 Department of Emergency, Emergency Medicine Unit, Geneva University Hospitals (HUG), 1211 Geneva, Switzerland; francois.sarasin@hcuge.ch

**Keywords:** COVID-19, fears, lockdown, post-lockdown, containment measures, isolation, hopelessness, loneliness, work status, dying, getting sick, young patients, elderly, psychiatric emergency department, mental health

## Abstract

*Background and Objectives*: While the impact on mental health of 2019 coronavirus (COVID-19) has been extensively documented, little is known about its influence on subjective fears. Here, we investigate the COVID-19 impact and its related restrictions on fears of patients admitted to a psychiatric Emergency Department (ED) during and post-lockdown. *Materials and Methods*: A retrospective study on 1477 consultations at the psychiatric ED of the University Hospital of Geneva (HUG) was performed using a mixed-methods analysis. The first analysis section was qualitative, aiming to explore the type of fears, while the second section statistically compared fears (i) during lockdown (16 March 2020–10 May 2020) and (ii) post-lockdown (11 May 2020–5 July 2020). Fears were also explored among different patient-age sub-groups. *Results*: 334 patients expressed one/more fears. Both in lockdown and post-lockdown, fears mostly pertained to “containment measures” (isolation, loneliness). When compared lockdown vs. post-lockdown, fears about “work status” (deteriorating, losing work) prevailed in lockdown (*p* = 0.029) while “hopelessness” (powerless feeling, inability to find solutions) in post-lockdown (*p* = 0.001). “Self around COVID-19” (dying, getting sick) fear was relatively more frequent in youth (*p* = 0.039), while “hopelessness” in the elderly (*p* < 0.001). *Conclusions*: Collectively, these findings highlight that lockdown/post-lockdown periods generated temporally and demographically distinct COVID-19 related fears patterns, with special regard to youth and elderly, two particularly vulnerable populations when faced with sudden and unexpected dramatic events. For this reason, the particular ED “front-line service” status makes it a privileged observatory that can provide novel insights. From a mental health perspective, these latter can be translated into pragmatic, more personalized prevention strategies to reinforce specific resilience resources and mitigate the current and long-term pandemic’s impact.

## 1. Introduction

The 2019 coronavirus disease (COVID-19) pandemic originated from pneumonia-like respiratory illnesses in Wuhan, China, in December 2019 [1]. Since then, it has rapidly spread throughout the world and was declared a public health emergency by the World Health Organization (WHO) by the end of January 2020. In Switzerland, the first cases of COVID-19 were detected in late February 2020, followed by the outbreak of several infection clusters throughout the country, resulting in a national lockdown, which was gradually lifted mid-May 2020 as the most alarming phase of the pandemic had passed [2,3].

The direct and indirect stressful effects of the COVID-19 lockdown and post-lockdown measures to restrict people’s movements and help curb the epidemic had a negative impact on mental health (particularly: post-traumatic stress symptoms, PTSD, anxiety, and depression) [4,5]. According to a recent review by Gianfredi and colleagues who examined several studies that had looked at internet searches across two dozen countries, there was a statistically significant increase in internet searches related to mental health issues during the COVID-19 pandemic. The most commonly used search terms were fear, stress, solitude, anxiety, depression, and suicide thoughts [6]. Similar to many other countries, the evolution of the pandemic and public health response in Switzerland also witnessed the rise of a secondary mental health crisis [7,8,9]. As the pandemic and societal responses to its course are constantly and rapidly evolving, the full impact of COVID-19 related socio-economic and healthcare upheavals on the general population is yet to be thoroughly investigated and fully understood.

A number of COVID-19 related fears in the general population have been described in the recent psychiatric literature. While fear is generally defined as an emotional response to a perceived threat, it can also lead to temporary physiological changes, emotional distress, and behavioral avoidance [10,11]. In the context of infectious diseases such as COVID-19, Schimmenti and colleagues suggested categorizing fear as follows [12]: (i) fear of/for one’s body, (ii) fear of and for significant others, (iii) fear of knowing and not knowing enough about the disease, and (iv) fear of taking action and of not being proactive enough. Other types of fears include fear of stigmatization for being a health care worker, a survivor of the disease, or a foreigner [10]. Several instruments have been developed to quantify the different types of fear associated with COVID-19 and assess their impact on the mental health of the general population and health care workers [13,14,15]. The fear of COVID-19 scale (The Fear of COVID-19 Scale, FCV-19S) is the most commonly used instrument at the moment, having been translated to several languages and validated against several mental health problems, including fear and anxiety [10].

Based on previous coronavirus infection (Severe Acute Respiratory Syndrome, SARS or Middle-Est Respiratory Syndrome, MERS) models, Torales and colleagues hypothesized that the COVID-19 related containment measures (isolation and confinement) might lead to aggravated mental health consequences, especially in individuals with prior mental health conditions, ranging from anxiety, fear, and depressive symptoms to psychophysical stress and functional neurological disorder [16].

Health care workers, in particular, are faced with the additional fear of transmitting the disease to relatives and may suffer from PTSD and other psychiatric symptoms [16,17,18]. In a meta-analysis involving nearly 2000 studies from several countries on psychiatric and neuropsychiatric presentations of individuals with suspected or laboratory-confirmed coronavirus infection (SARS, MERS, or SARS-CoV-2), the authors concluded that most COVID-19 patients should recover without experiencing mental illness [19], although a significant proportion will suffer from delirium in the acute stage and may experience depression, anxiety, fatigue, PTSD, and rarer neuropsychiatric syndromes in the long-term. In a recent analysis of how British print media reported on COVID-19-related grief and bereavement, Sowden and colleagues found a rather homogeneous fear-based narrative that was lacking in nuance [20], often resorting to sensationalist language to stir fears, war metaphors to relate a growing uncertainty and fear of the future, or euphemistic (pass away) or glorifying (heroes) language to report on personal tragedies of bereavement and grief. As language can alter how individuals perceive events, fear, and anxiety, in their most heightened forms, it can render them dysfunctional. Presti and colleagues suggested that the current pandemic led to heightened anxiety and fear, disruption to the sense of self, as well as prejudices and discrimination [11]. Furthermore, they argued that psychological flexibility, i.e., the ability to shift one’s mindset and adapt to new circumstances, may allow individuals to maintain balance and avoid dysfunction.

Nevertheless, studies on the composition of the COVID-19 related fears in the general population as well as those highly vulnerable to psychiatric complications during the pandemic are still scarce. Therefore, the objective of this study is to characterize all the different types of COVID-19 related fears in patients admitted at the psychiatric Emergency Department (ED) of the University Geneva Hospital (HUG) during lockdown and post lockdown in order to better understand the impact of the pandemic and its restrictive measures on subjective experiences.

## 2. Materials and Methods

### 2.1. Study Subjects

A retrospective analysis of all the consultations carried out at the psychiatric ED of the HUG, Switzerland, between 16 March and 5 July 2020 was conducted. All patients who were ≥16 years of age (the age limit for admission in this service) with a psychiatric medical evaluation, were included. Informed consent was waived to avoid selection bias. Gender, current age, familial and residential status, urgency degree, and psychiatric diagnosis at admission were analyzed per previously established methodologies in the same department and institution [21,22]. Urgency degree was categorized according to the Echelle Suisse du Tri (EST^®^) (HUG, Switzerland), which includes four levels of urgency: 1—urgent, life-threatening condition; 2—pathological, non-life-threatening but with the potential to rapidly deteriorate; 3—pathological but not time-sensitive (e.g., the patient arrives in a stable condition); and 4—medically stable and not requiring urgent care. We have chosen a psychiatric ED because, as a front-line service, it is precociously representative of the mental health well-being of the population [21,23].

### 2.2. Ethical Concerns

The Research Ethics Committee of Geneva (Switzerland) reviewed and approved all study procedures under the registration number 2020-01510 (approval date: 29 June 2020). The study design was conducted in accordance with the guidelines provided in the current version of the Declaration of Helsinki as revised in 2013 [24].

### 2.3. Data Analysis

The study utilized a mixed-method analysis. The first part of the analysis was qualitative, aiming to explore the presence and type of COVID-19 fears, while the second part compared fears statistically during two periods: (i) during lockdown (16 March 2020–10 May 2020) and (ii) post-lockdown periods (11 May 2020–5 July 2020) (please see [8] for how these exact dates were chosen). Moreover, these fears were explored for different patient-age sub-groups. We thus have two separate patient subgroups (lockdown and post-lockdown) based on the date of their consultation.

#### 2.3.1. Qualitative Assessment

Patients were not asked predefined questions but were able to spontaneously express their fears related to COVID-19. This approach was chosen to examine, in an exploratory way, the fears that emerged without influencing or conditioning them in any way. Qualitative assessment was applied according to the Thematic Analysis proposed by Braun and Clarke [25], aimed to identify patterns (themes) within data. According to this methodology, themes were inductively derived from the data rather than established in advance or fitted into a pre-existing frame or theory [25,26]. This approach is similar to the Interpretative Phenomenological Analysis (IPA) used by Smith [27,28]. However, the Thematic Analysis did not originate from a specific epistemological position, an element that provides it with greater flexibility, including the possibility of assigning percentage values while maintaining internal consistency and coherence [29]. Still, we attempted to select the themes such that (i) our results were easily comparable to what had already been examined in the literature and (ii) conductive to the purpose of this study, namely, to provide insight and guidance for the development of novel and more specific mental health strategies, e.g., with regard to a patient’s age. Three independent raters (JA, AF, LM) examined all consultation transcripts, which contained verbatim patient statements and detailed descriptions by the consultant, for the identification of themes. Findings were compared and yielded an average agreement of 90% between rates. Possible disagreements were resolved through a discussion with the senior researcher (AC), upon which the themes were defined by consensus. In consideration of this large sample size, percentage values representing the prevalence of the main themes were also provided. For the coding, we employed the Delve qualitative data analysis tool (Twenty to Nine LLC, USA).

#### 2.3.2. Statistical Assessment

Distributions of each type of fear were computed. Continuous and categorical variables were represented as mean and standard deviation (SD) and number and percentage, respectively. The normal distribution of our sample was confirmed by the Kolmogorov-Smirnov test. Afterward, the total sample was divided according to the admission dates at HUG ED into two groups, admitted during the lockdown period and post-lockdown period. As lockdown measures in Switzerland were implemented in the absence of a strict stay-at-home order, the formal definition of lockdown may be different elsewhere. We based the definition on the timeline of the school closure and reopening. As such, patients admitted from 16 March 2020 to 10 May 2020 were placed in the lockdown group, and patients with admission between 11 May 2020 and 5 July 2020 were placed in the post-lockdown group. In each of these two groups, three additional subgroups were defined according to age: young (16–25 years old), adult (26–64 years old), and elderly subjects (≥65 years old). Pearson’s chi-square test with Yate correction was performed to evaluate potential differences among concerns related to COVID-19 infection. All statistical analyses were conducted using the Statistical Package for Social Sciences (version 25.0, SPSS; SPSS Inc., Chicago, IL, USA) for Windows, and the significance threshold was set at *p* < 0.05 (two-tailed).

## 3. Results

### 3.1. Socio-Demographic and Clinical Characteristics of the Participants Who Expressed One or more COVID-19 Related Fears

The total sample consisted of 1477 consultations (*n* = 688 during lockdown and *n* = 809 post-lockdown). In some of these 1477 consultations (*n* = 334, 22.6%), patients expressed one or more COVID-19 related fears, the mean ± SD age was 42.77 ± 17.37 years, the majority (*n* = 180, 53.9%) were females, and nearly half of the subjects included (*n* = 163, 48.8%) were unmarried/not in a relationship. All 334 patients who expressed a COVID-19-related fear stated that they had been adhering to the public confinement orders, although those were less severe in Switzerland compared to other countries (see above). The majority of patients (*n* = 295, 88.3%) had come from their private residence. The most represented urgency degree was 2 (*n* = 147, 44.0%). The most represented psychiatric diagnosis was depression/anxiety (*n* = 110, 32.9%), followed by suicidal ideation (SI) and suicidal attempts (SA) (*n* = 81, 24.3%) (Table 1).

### 3.2. Qualitative and Statistical Results: COVID-19 Related Fears in General, According to the Date of Admission, and according to Different Age Groups

In both lockdown and post-lockdown, the most represented fear theme pertained to isolation, loneliness, and other difficulties related to “containment measures” “having to stay at home, loneliness, isolation” (*n* = 230, 68.9%), followed by fear for “self around COVID-19” (fear of dying, fear of getting sick) (*n* = 53, 15.9%) (Table 2).

When the comparison was performed according to the date of admission, fears related to “containment measures” remained the most frequent during both the lockdown and post-lockdown periods (*n* = 79, 65.3% and *n* = 151, 70.9%, respectively). The second most prevalent fear theme pertained to “self around COVID-19” (*n* = 25, 20% during lockdown period and *n* = 28, 13.1% during post-lockdown period). However, there are no significant differences in the prevalence of these fears as the lockdown measures were eased. Notably, “hopelessness” (powerless feeling in the face of the current situation and not being able to find solutions) was significantly more frequent in the post-lockdown period than in the lockdown period (*n* = 12, 5.6% vs. *n* = 1, 0.8%, *p* = 0.029). Conversely, the fear about the “work status” (losing or deteriorating work status) was more frequently reported by patients admitted during the lockdown period than in the post-lockdown period (*n* = 15, 12.4% vs. *n* = 7, 3.3%, *p* = 0.001) (Table 2).

In this context, it is also interesting to note that while overall admissions were only about 21% higher in post-lockdown compared to the lockdown period (see Table 1 in [8]), the expression of COVID-related fears was about 81% higher during post-lockdown.

When the comparison was performed across different age groups, young patients reported a significant increase in fear for “self around COVID-19” (*n* = 58, 25.3%) in comparison to those from adult (*n* = 30, 13.1%) and elderly subjects (*n* = 4, 13.3%) (*p* = 0.039). Lastly, elderly patients reported a significant increase in “hopelessness” (*n* = 5, 16.7%) compared to young (*n* = 0, 0.0%) and adult patients (*n* = 8, 3.5%) (*p* < 0.001) (Table 3).

## 4. Discussion

To the best of our knowledge, this is the first study that characterizes the fears of patients admitted to a psychiatric ED during different time periods of the COVID-19 pandemic.

Our work revealed that fears related to containment measures with their psychological impact (loneliness and isolation) and those related to the safety of oneself in regard to SARS-CoV-2 infection are the major fears. These findings are in line with a large-scale study conducted in several European countries with varying degrees of infection burden, in which participants reported a pronounced sense of loneliness and fear of COVID-19 [30]. Interestingly, in our study, these concerns persist throughout the evolution of the COVID-19 pandemic, regardless of changes in social sanitary measures. On the contrary, a previous study of more than 200,000 participants across the US and Europe has shown that COVID-related fears gradually declined during the post-lockdown phase [31].

Surprisingly, we did not detect any fears of spreading the infection to another individual. Our findings contrast with previous reports of COVID-19 related concerns in healthcare professionals [16,17,32,33], which documented a high level of mental distress in nurses, doctors, and hospital staff who were at high risk of COVID-19 contamination and, consequentially, concerned about the possibility of unknowingly spreading the infection to family members, patients, and coworkers [34]. Healthcare professionals also reported that this latter fear compromised their attention and decision-making [35]. These discrepancies might be attributed to the clinical and demographic characteristics of the study subjects as we only had patients that were acutely admitted to psychiatric ED [36]. Therefore, in case of fears related to containment measures, the psychological imprint from the lockdown period might be more long-lasting in this group of highly vulnerable individuals. In fact, Varga et al. also demonstrated that individuals with a history of mental illness were more affected by COVID-19 related concerns [31]. Similarly, fears for the safety of others might not be the most urgent matter for patients in psychiatric ED, whose mental health status is already experiencing deterioration and/or is at imminent risk of further decline.

Our work also documented an increase in fear about employment status during the lockdown period, while the experience of hopelessness became more prominent during the post-lockdown phase. During the lockdown in Switzerland, personal and public businesses, entertainment venues, and schools were not allowed to operate. As a consequence, these restrictive measures created unprecedented economic pressure on the general population with the emergence of COVID-19 related unemployment and bankruptcy. While economic stimuli were introduced by the Swiss Federal Council in Spring 2020 to mitigate these hardships [37], some of the most vulnerable entities (small businesses, gig workers, seasonal and migrant workers) are expected to be unable to recover from the pandemic. As such, a sense of hopelessness coupled with anxiety for an uncertain future is expected in the affected individuals even as the lockdown measures were eased, while the predominant concern throughout the lockdown is directly related to the loss of employment due to the closure of the economy. These findings have important implications for the strategic design and implementation of socio-economic policies in response to future catastrophes, with an emphasis on supporting initiatives for the sectors of society that have a low probability of recovery.

The youth and elderly are among the most vulnerable in society during the pandemic, and previous studies have documented various mental health fears for these demographic groups [38,39]. Our study revealed that fears about getting sick or dying from COVID-19 are prevalent in younger patients. Interestingly, a study conducted in Poland including more than 1000 participants showed that the most prominent concerns in youths are related to COVID-19 restrictive social measures [40]. This discrepancy might be related to differences in the lockdown policies between Switzerland and Poland. The former did not implement a strict lockdown with stay-at-home orders, and the sanitary measures were swiftly lifted in May 2020 [41]. In contrast, Poland had a more prolonged lockdown with strictly monitored stay-at-home provisions [42]. With regard to fears among the elderly during the COVID-19 pandemic, different observations have been documented worldwide. A study conducted in Bangladesh showed that fear of getting infected prevails in this age group while the elderly in the United States (US) experienced more fears about isolation and loneliness [43,44]. The Swiss elderly in this study showed a prominent sense of hopelessness, which is consistent with previous observations made in elderly with a comorbid physical illness admitted to a psychiatric ED [45] or suffering from a neurological disorder [46,47,48]. These differences could be explained in terms of the socio-economic disparity between developing countries, where crowded living conditions heighten the fear of contagion, and the US and Europe, where adequate isolation is possible but may inadvertently generate a sense of extreme isolation, loneliness, and hopelessness about the cessation of the pandemic and return to normal life.

This study needs to be interpreted in light of several limitations. Firstly, we did not employ validated questionnaires but analyzed what spontaneously emerged from the patients’ discourses. At the same time, this was the result of a specific methodological choice (see Methods section). Secondly, this report focuses on consultations made in Switzerland and, as discussed above, several socio-economic and demographic factors need to be taken into consideration in order to correctly interpret the findings. Thirdly, lockdown measures in Switzerland were more relaxed as they did not involve explicit stay-at-home orders. Therefore, this more lenient policy may have generated different COVID-19 lockdown impacts in Switzerland, especially with regard to the clinical features of psychiatric ED admissions. Fourthly, these findings are associated with the initial lockdown event and level of COVID-19 infections at that time, hence a more systematic analysis of the different phases of the pandemic may be required to generate broader insights regarding the chronic impact of lockdowns on mental health. Fifthly, it is difficult to obtain reliable statements and self-assessments from patients with acute psychotic symptoms. Nevertheless, only a few patients had a psychiatric diagnosis of “psychotic episode” (*n* = 11, 3.3%), while the psychiatric diagnosis of “depression/anxiety” was much more common (*n* = 110, 32.9%) and represented the largest group (see Table 1). The effect of this limitation on the overall results should therefore be rather small. Sixthly, we neglected the potential effects of concomitant psychopharmacological treatments or psychotherapies that could potentially affect the fears expressed by patients. Finally, the association with the psychiatric diagnosis was not investigated. Thus, a more informative picture of COVID-19-related fears, including possible inferred observations from this latter aspect, could not be obtained.

Other factors that we did not take into account but that could be considered in future studies are the duration and types of confinement, personal COVID-19 situation (contagion, infected relatives, vaccination status, etc.), previous experience with infectious diseases, previous consultations/hospitalization and/or existence of a previous psychiatric (chronic) diagnosis, type of pharmacological treatment, etc., which each could give rise to new subgroup comparisons.

A major element of novelty of our study lies in its examination of the particular population of patients suffering from an acute mental health crisis that requires psychiatric ED admission. As such, the work is expected to provide novel insights about subjective fears for the prevention of mental health consequences in this particularly vulnerable population during the pandemic [49]. Finally, these findings could be used to create a new questionnaire about COVID-19-related fears and contribute to already existing ones.

## 5. Conclusions

Our study describes the COVID-19-related fears during the lockdown and post-lockdown periods among patients admitted to psychiatric ED in Geneva. Collectively, reported findings highlight that lockdown and post-lockdown periods generated temporally and demographically distinct patterns of COVID-19 related fears, with special regard to youth and elderly, two vulnerable populations faced catastrophes. In light of this, the particular ED “front-line service” status makes it a privileged observatory that can provide novel insights. From a mental health perspective, these latter can be translated into appropriate and pragmatic preventive interventions, specifically tailored to the affected populations, in order to reinforce specific resilience resources and mitigate the impact of the current pandemic and its possible long-term effects [4,5,21,50,51,52,53,54].

## Figures and Tables

**Table 1:** Socio-demographic and clinical characteristics of patients who expressed one or more COVID-19 related fears (*n* = 334).

Socio-Demographic and Clinical Characteristics	*n* (%)
Female gender, *n* (%)	180 (53.9)
Current age, in years, mean ± SD	42.77 ± 17.37
Familial status, *n* (%)	
Unmarried/not in relationship	163 (48.8)
Married/in relationship	90 (26.9)
Separated/divorced	66 (19.8)
Widowed	15 (4.5)
Residential status, *n* (%)	
Private residence	295 (88.3)
Foster home, hotel	30 (8.9)
Homeless	6 (1.8)
Migrant	3 (0.9)
Urgency degree, according to EST^®^, *n* (%)	
Degree 1	71 (21.3)
Degree 2	147 (44.0)
Degree 3	97 (29.0)
Degree 4	19 (5.7)
Psychiatric diagnosis, *n* (%)	
Psychotic Episode	11 (3.3)
Manic/hypomanic episode	6 (1.8)
Depression/anxiety	110 (32.9)
Suicidal ideation and behavior	81 (24.3)
Substance use disorder	19 (5.7)
Behavioral disorder (among adults and elderly)	36 (10.8)
Psychomotor agitation	28 (8.4)
Somatic problem	38 (11.4)
Others	5 (1.5)

**Notes:** Urgency degree according to EST^®^: degree 1—a very urgent life-threatening condition; degree 2—a non-life-threatening pathological situation that is likely to worsen quickly; degree 3—a pathological situation where time is not a critical factor, and the patient is stable upon arrival; and degree 4—a stable medical condition that does not require emergency care. Abbreviations: EST, Echelle Suisse du Tri (EST^®^) (HUG, Switzerland).

**Table 2:** Differences in COVID-19 related fears according to admission periods (lockdown vs. post-lockdown). For a general comparison of the socio-demographic characteristics between the lockdown/post-lockdown groups, please see Table 1 in [8].

Fear Theme	Total Sample: *Total n* = 334 *n,* (%)	Lockdown: *Total n* = 121 *n,* (%)	Post- Lockdown: *Total n* = 213 *n,* (%)	Chi^2^	*p*
Self around COVID-19 (fear of dying, fear of getting sick)	53 (15.9)	25 (20.7)	28 (13.1)	3.265	0.071
Fear for loved ones (fear of parents getting sick, etc)	24 (7.2)	8 (6.6)	16 (7.5)	0.094	0.759
Fears related to containment measures (having to stay at home, isolation, loneliness)	230 (68.9)	79 (65.3)	151 (70.9)	1.130	0.288
Fear of being stigmatized (in case of illness or suspicion of illness)	2 (0.6)	0 (0.0)	2 (0.9)	1.143	0.285
Fear related to hopelessness (powerless feeling in the face of the current situation, not being able to find solutions)	13 (3.9)	1 (0.8)	12 (5.6)	4.767	0.001 *
Fear of demoralization feeling because of COVID-19 and its actual/potential impact on the individual	23 (6.9)	8 (6.6)	15 (7.0)	0.022	0.881
Fear about work status (losing work or deteriorating work status)	22 (6.6)	15 (12.4)	7 (3.3)	10.409	0.029 *
Fear of not being helped by others in times of need	1 (0.3)	0 (0.0)	1 (0.5)	0.570	0.450
Other	36 (10.8)	12 (9.9)	24 (11.3)	0.702	0.702

**Legend:** * statistically significant (to *p* < 0.05).

**Table 3:** Differences in COVID-19 related fears according to different age groups.

Fear Theme	Young Subjects (16–25 Years Old) *n* = 75	Adult Subjects (26–64 Years Old) *n* = 229	Elderly Subjects (≥65 Years Old) *n* = 30	Chi^2^	*p*
Self around COVID-19 (fear of dying, fear of getting sick)	19 (25.3)	30 (13.1)	4 (13.3)	6.491	0.039 *
Fear for loved ones (fear of parents getting sick, etc)	5 (6.7)	17 (7.4)	2 (6.7)	0.062	0.970
Fears related to containment measures (having to stay at home, isolation, loneliness)	52 (69.3)	159 (69.4)	19 (63.3)	0.470	0.791
Fear of being stigmatized (in case of illness or suspicion of illness)	0 (0.0)	1 (0.4)	1 (3.3)	4.322	0.115
Fear related to hopelessness (powerless feeling in the face of the current situation, not being able to find solutions)	0 (0.0)	8 (3.5)	5 (16.7)	16.222	<0.001 *
Fear of demoralization feeling because of COVID-19 and its actual/potential impact on the individual	7 (9.3)	16 (7.0)	0 (0.0)	2.923	0.232
Fear about work status (losing work or deteriorating work status)	4 (5.3)	17 (7.4)	1 (3.3)	0.968	0.616
Fear of not being helped by others in times of need	0 (0.0)	1 (0.4)	0 (0.0)	0.460	0.795
Other	6 (8.0)	25 (10.9)	5 (16.7)	1.688	0.430

**Legend:** * statistically significant (to *p* < 0.05).

## Data Availability

The datasets generated for this study are available on request to the corresponding author.

## References

[1] World Health Organization Flambée de Maladie à Coronavirus 2019 (COVID-19). https://www.who.int/fr/emergencies/diseases/novel-coronavirus-2019.

[2] Federal Office of Public Health New Coronavirus 2019-nCoV: First Confirmed Case in Switzerland. https://www.bag.admin.ch/bag/en/home/das-bag/aktuell/medienmitteilungen.msg-id-78233.html.

[3] Giachino M., Valera C.B.G., Rodriguez Velásquez S., Dohrendorf-Wyss M.A., Rozanova L., Flahault A. (2020). Understanding the dynamics of the COVID-19 pandemic: A real-time analysis of Switzerland’s first wave. Int. J. Environ. Res. Public Health.

[4] Fiorillo A., Gorwood P. (2020). The consequences of the COVID-19 pandemic on mental health and implications for clinical practice. Eur. Psychiatry.

[5] Costanza A., Di Marco S., Burroni M., Corasaniti F., Santinon P., Prelati M., Chytas V., Cedraschi C., Ambrosetti J. (2020). Meaning in life and demoralization: A mental-health reading perspective of suicidality in the time of COVID-19. Acta Biomed..

[6] Gianfredi V., Provenzano S., Santangelo O.E. (2021). What can internet users’ behaviours reveal about the mental health impacts of the COVID-19 pandemic? A systematic review. Public Health.

[7] Généreux M., Schluter P.J., Hung K.K., Wong C.S., Pui Yin Mok C., O’Sullivan T., David M.D., Carignan M.E., Blouin-Genest G., Champagne-Poirier O. (2020). One virus, four continents, eight countries: An interdisciplinary and international study on the psychosocial impacts of the COVID-19 pandemic among adults. Int. J. Environ. Res. Public Health.

[8] Ambrosetti J., Macheret L., Folliet A., Wullschleger A., Amerio A., Aguglia A., Serafini G., Prada P., Kaiser S., Bondolfi G. (2021). Psychiatric emergency admissions during and after COVID-19 lockdown: Short-term impact and long-term implications on mental health. BMC Psychiatry.

[9] Puccinelli P.J., Costa T.S., Seffrin A., de Lira C.A.B., Vancini R.L., Knechtle B., Nikolaidis P.T., Andrade M.S. (2021). Physical activity levels and mental health during the COVID-19 pandemic: Preliminary results of a comparative study between convenience samples from Brazil and Switzerland. Medicina.

[10] Muller A.E., Himmels J.P.W., Van de Velde S. (2021). Instruments to measure fear of COVID-19: A diagnostic systematic review. BMC Med. Res. Methodol..

[11] Presti G., Mchugh L., Gloster A., Karekla M., Hayes S.C. (2020). The dynamics of fear at the time of covid-19: A contextual behavioral science perspective. Clin. Neuropsychiatry.

[12] Schimmenti A., Billieux J., Starcevic V. (2020). The four horsemen of fear: An integrated model of understanding fear experiences during the COVID-19 pandemic. Clin. Neuropsychiatry.

[13] Ahorsu D.K., Lin C.Y., Imani V., Saffari M., Griffiths M.D., Pakpour A.H. (2020). The fear of COVID-19 scale: Development and initial validation. Int. J. Ment. Health Addict..

[14] Tzur Bitan D., Grossman-Giron A., Bloch Y., Mayer Y., Shiffman N., Mendlovic S. (2020). Fear of COVID-19 scale: Psychometric characteristics, reliability and validity in the Israeli population. Psychiatry Res..

[15] Schimmenti A., Starcevic V., Giardina A., Khazaal Y., Billieux J. (2020). Multidimensional assessment of COVID-19-related fears (MAC-RF): A theory-based instrument for the assessment of clinically relevant fears during pandemics. Front. Psychiatry.

[16] Torales J., O’Higgins M., Castaldelli-Maia J.M., Ventriglio A. (2020). The outbreak of COVID-19 coronavirus and its impact on global mental health. Int. J. Soc. Psychiatry.

[17] Amerio A., Bianchi D., Santi F., Costantini L., Odone A., Signorelli C., Costanza A., Serafini G., Amore M., Aguglia A. (2020). Covid-19 pandemic impact on mental health: A web-based cross-sectional survey on a sample of Italian general practitioners. Acta Biomed..

[18] Aguglia A., Amerio A., Costanza A., Parodi N., Copello F., Serafini G., Amore M. (2021). Hopelessness and post-traumatic symptoms among healthcare workers during the Covid-19 pandemic: Any role for mediating variables?. Int. J. Environ. Res. Public Health.

[19] Rogers J.P., Chesney E., Oliver D., Pollak T.A., McGuire P., Fusar-Poli P., Zandi M.S., Lewis G., David A.S. (2020). Psychiatric and neuropsychiatric presentations associated with severe coronavirus infections: A systematic review and meta-analysis with comparison to the COVID-19 pandemic. Lancet Psychiatry.

[20] Sowden R., Borgstrom E., Selman L.E. (2021). ‘It’s like being in a war with an invisible enemy’: A document analysis of bereavement due to COVID-19 in UK newspapers. PLoS ONE.

[21] Costanza A., Mazzola V., Radomska M., Amerio A., Aguglia A., Prada P., Bondolfi G., Sarasin F., Ambrosetti J. (2020). Who consults an adult psychiatric emergency department? Pertinence of admissions and opportunities for telepsychiatry. Medicina.

[22] Ambrosetti J., Macheret L., Folliet A., Wullschleger A., Amerio A., Aguglia A., Serafini G., Prada P., Kaiser S., Bondolfi G. (2021). Impact of the COVID-19 pandemic on psychiatric admissions to a large swiss emergency department: An observational study. Int. J. Environ. Res. Public Health.

[23] Ostertag L., Golay P., Dorogi Y., Brovelli S., Bertran M., Cromec I., Van Der Vaeren B., Khan R., Costanza A., Wyss K. (2019). The implementation and first insights of the French-speaking Swiss programme for monitoring self-harm. Swiss. Med. Wkly..

[24] World Medical Association (2013). World Medical Association Declaration of Helsinki: Ethical principles for medical research involving human subjects. JAMA.

[25] Braun V., Clarke V. (2006). Using thematic analysis in psychology. Qual. Res. Psychol..

[26] Costanza A., Amerio A., Odone A., Baertschi M., Richard-Lepouriel H., Weber K., Di Marco S., Prelati M., Aguglia A., Escelsior A. (2020). Suicide prevention from a public health perspective. What makes life meaningful? The opinion of some suicidal patients. Acta Biomed..

[27] Smith J.A., Osborn M., Smith J.A. (2003). Interpretative phenomenological analysis. Qualitative Psychology: A Practical Guide to Methods.

[28] Smith J.A., Flowers P., Larkin M. (2009). Interpretative Phenomenological Analysis: Theory, Method and Research.

[29] Holloway I., Todres L. (2003). The status of the method: Flexibility, consistency and coherence. Qual. Res..

[30] Lo Coco G., Gentile A., Bosnar K., Milovanović I., Bianco A., Drid P., Pišot S. (2021). A cross-country examination on the fear of covid-19 and the sense of loneliness during the first wave of COVID-19 outbreak. Int. J. Environ. Res. Public Health.

[31] Varga T.V., Bu F., Dissing A.S., Elsenburg L., Bustamante J.J.H., Matta J., van Zon S.K.R., Brouwer S., Bültmann U., Fancourt D. (2021). Loneliness, worries, anxiety, and precautionary behaviours in response to the COVID-19 pandemic: A longitudinal analysis of 200,000 Western and Northern Europeans. Lancet Reg. Health Eur..

[32] Britt T.W., Shuffler M.L., Pegram R.L., Xoxakos P., Rosopa P.J., Hirsh E., Jackson W. (2021). Job demands and resources among healthcare professionals during virus pandemics: A review and examination of fluctuations in mental health strain during COVID-19. Appl. Psychol..

[33] De Kock J.H., Latham H.A., Leslie S.J., Grindle M., Munoz S.A., Ellis L., Polson R., O’Malley C.M. (2021). A rapid review of the impact of COVID-19 on the mental health of healthcare workers: Implications for supporting psychological well-being. BMC Public Health.

[34] Berardelli I., Vaia A., Pompili M. (2021). Thoughts of death, depression, and guilt in a healthcare worker who infected her husband with SARS-CoV-2: A case report. CNS Neurol. Disord. Drug. Targets.

[35] Kang L., Li Y., Hu S., Chen M., Yang C., Yang B.X., Wang Y., Hu J., Lai J., Ma X. (2020). The mental health of medical workers in Wuhan, China dealing with the 2019 novel coronavirus. Lancet Psychiatry.

[36] Baertschi M., Costanza A., Canuto A., Weber K. (2018). The Function of Personality in Suicidal Ideation from the Perspective of the Interpersonal-Psychological Theory of Suicide. Int. J. Environ. Res. Public Health.

[37] KPMG Switzerland: Government and Institution Measures in Response to COVID-19. https://home.kpmg/xx/en/home/insights/2020/04/switzerland-government-and-institution-measures-in-response-to-covid.html.

[38] Xu Q., Mao Z., Wei D., Liu P., Fan K., Wang J., Wang X., Lou X., Lin H., Wang C. (2021). Prevalence and risk factors for anxiety symptoms during the outbreak of COVID-19: A large survey among 373216 junior and senior high school students in China. J. Affect. Disord..

[39] Schweda A., Weismüller B., Bäuerle A., Dörrie N., Musche V., Fink M., Kohler H., Teufel M., Skoda E.M. (2021). Phenotyping mental health: Age, community size, and depression differently modulate COVID-19-related fear and generalized anxiety. Compr. Psychiatry.

[40] Gambin M., Sękowski M., Woźniak-Prus M., Wnuk A., Oleksy T., Cudo A., Hansen K., Huflejt-Łukasik M., Kubicka K., Łyś A.E. (2021). Generalized anxiety and depressive symptoms in various age groups during the COVID-19 lockdown in Poland. Specific predictors and differences in symptoms severity. Compr. Psychiatry.

[41] Sager F., Mavrot C. (2020). Switzerland’s COVID-19 policy response: Consociational crisis management and neo-corporatist reopening. Eur. Policy Anal..

[42] Nazar W., Leszkowicz J., Pieńkowska A., Brzeziński M., Szlagatys-Sidorkiewicz A., Plata-Nazar K. (2020). Before-and-after online community survey on knowledge and perception of COVID-19 pandemic. BMC Infect. Dis..

[43] Peng S., Roth A.R. (2021). Social isolation and loneliness before and during the COVID-19 pandemic: A longitudinal study of US Adults over 50. J. Gerontol. B Psychol. Sci. Soc. Sci..

[44] Mistry S.K., Ali A.R.M.M., Akther F., Yadav U.N., Harris M.F. (2021). Exploring fear of COVID-19 and its correlates among older adults in Bangladesh. Glob. Health.

[45] Costanza A., Amerio A., Radomska M., Ambrosetti J., Di Marco S., Prelati M., Aguglia A., Serafini G., Amore M., Bondolfi G. (2020). Suicidality assessment of the elderly with physical illness in the emergency department. Front. Psychiatry.

[46] Costanza A., Amerio A., Aguglia A., Escelsior A., Serafini G., Berardelli I., Pompili M., Amore M. (2020). When sick brain and hopelessness meet: Some aspects of suicidality in the neurological patient. CNS Neurol. Disord. Drug. Targets.

[47] Costanza A., Baertschi M., Weber K., Canuto A. (2015). Maladies neurologiques et suicide: De la neurobiologie au manque d’espoir [Neurological diseases and suicide: From neurobiology to hopelessness]. Rev. Med. Suisse.

[48] Costanza A., Xekardaki A., Kövari E., Gold G., Bouras C., Giannakopoulos P. (2012). Microvascular burden and Alzheimer-type lesions across the age spectrum. J. Alzheimers Dis..

[49] Amerio A., Aguglia A., Odone A., Gianfredi V., Serafini G., Signorelli C., Amore M. (2020). COVID-19 pandemic impact on mental health of vulnerable populations. Acta Biomed..

[50] Costanza A., Ambrosetti J., Wyss K., Bondolfi G., Sarasin F., Khan R.A. (2018). Prévenir le suicide aux urgences : De la “Théorie Interpersonnelle du Suicide” à la *connectedness* [Prevention of suicide at Emergency Room: From the “Interpersonal Theory of Suicide” to the connectedness]. Rev. Med. Suisse.

[51] Maulik P.K., Thornicroft G., Saxena S. (2020). Roadmap to strengthen global mental health systems to tackle the impact of the COVID-19 pandemic. Int. J. Ment. Health Syst..

[52] Osimo S.A., Aiello M., Gentili C., Ionta S., Cecchetto C. (2021). The influence of personality, resilience, and alexithymia on mental health during COVID-19 pandemic. Front. Psychol..

[53] Costanza A., Baertschi M., Richard-Lepouriel H., Weber K., Berardelli I., Pompili M., Canuto A. (2020). Demoralization and Its Relationship with Depression and Hopelessness in Suicidal Patients Attending an Emergency Department. Int. J. Environ. Res. Public Health.

[54] Costanza A., Baertschi M., Richard-Lepouriel H., Weber K., Pompili M., Canuto A. (2020). The Presence and the Search Constructs of Meaning in Life in Suicidal Patients Attending a Psychiatric Emergency Department. Front. Psychiatry.

